# The Identification of Manure Spreading on Bare Soil through the Development of Multispectral Indices from Sentinel-2 Data: The Emilia-Romagna Region (Italy) Case Study

**DOI:** 10.3390/s24144687

**Published:** 2024-07-19

**Authors:** Marco Dubbini, Maria Belluzzo, Villiam Zanni Bertelli, Alessandro Pirola, Antonella Tornato, Cinzia Alessandrini

**Affiliations:** 1 Department of History and Cultures (DiSCi)—Geography Section, University of Bologna, Via Guerrazzi 20, 40125 Bologna, Italy; maria.belluzzo2@unibo.it (M.B.); ext-vzanni@arpae.it (V.Z.B.); 2 Arpae—Struttura IdroMeteoClima, Viale Silvani 6, 40122 Bologna, Italy; apirola@arpae.it (A.P.); calessandrini@arpae.it (C.A.); 3 Italian Institute for Environmental Protection and Research (ISPRA), Via Vitaliano Brancati 48, 00144 Roma, Italy; antonella.tornato@isprambiente.it

**Keywords:** remote sensing, Sentinel-2, manure spreading monitoring, spectral index, soil fertilisation, agricultural practices, assessment and validation, manure spectral index—MSI

## Abstract

Satellite remote sensing is currently an established, effective, and constantly used tool and methodology for monitoring agriculture and fertilisation. At the same time, in recent years, the need for the detection of livestock manure and digestate spreading on the soil is emerging, and the development of spectral indices and classification processes based on satellite multispectral data acquisitions is growing. However, the application of such indicators is still underutilised and, given the polluting impact of livestock manure and digestate on soil, groundwater, and air, an in-depth study is needed to improve the monitoring of this practice. Additionally, this paper aims at exposing a new spectral index capable of detecting the land affected by livestock manure and digestate spreading. This indicator was created by studying the spectral response of bare soil and livestock manure and digestate, using Copernicus Sentinel-2 MSI satellite acquisitions and ancillary datasets (e.g., soil moisture, precipitation, regional thematic maps). In particular, time series of multispectral satellite acquisitions and ancillary data were analysed, covering a survey period of 13 months between February 2022 and February 2023. As no previous indications on fertilisation practices are available, the proposed approach consists of investigating a broad-spectrum area, without investigations of specific test sites. A large area of approximately 236,344 hectares covering three provinces of the Emilia-Romagna Region (Italy) was therefore examined. A series of ground truth points were also collected for assessing accuracy by filling in the confusion matrix. Based on the definition of the spectral index, a value of the latter greater than three provides the most conservative threshold for detecting livestock manure and digestate spreading with an accuracy of 62.53%. Such results are robust to variations in the spectral response of the soil. On the basis of these very encouraging results, it is considered plausible that the proposed index could improve the techniques for detecting the spreading of livestock manure and digestate on bare ground, classifying the areas themselves with a notable saving of energy compared to the current investigation methodologies directly on the ground.

## 1. Introduction

Livestock manure and digestate management (identified henceforth by the acronym LMD), especially in relation to soil fertilisation practices, is responsible for the majority of polluting emissions in agriculture. These include ammonia (NH_3_) emissions, which, together with other forms of nitrogen such as nitrate ions (NO^3−^), play a fundamental role in environmental issues, affecting water, soil and air quality [1]. In the Emilia-Romagna region, the Regional Regulation No. 3 of 15 December 2017 governs the agricultural use of LMD and wastewater [2]. Rational and effective fertilisation, in compliance with good agricultural practices, keeps the amount of nitrogen in the soil-crop system in balance. In order to avoid the release of nitrogen into surface and groundwater, the use of livestock manure, digestate, and nitrogen fertilisers in the autumn–winter season is regulated by banning periods, from 1 November to 28 February [2].

Furthermore, according to the Regional Regulation [2], in order to contain the release of nitrogen from the soil to the water and the emission of ammoniacal nitrogen and foul odours into the atmosphere, the distribution techniques and other measures adopted must ensure, in particular, the uniform application of the fertiliser and its incorporation into the soil within twenty-four hours of the fertiliser being applied. Cultivated plots with vegetation cover in place and also those that have already been sown are excluded from these arrangements [2].

Companies that use LMD and other nitrogenous fertilisers are obliged to draw up an Agronomic Manure Use Plan (PUA), specifying the characteristics and size of the farm; the storage capacity; available areas, crops, and periods of LMD distribution [3].

They are also required to record individual distributions, either on plain paper or digitally, within fifteen days of the intervention. The legal representative of the farm must keep the aforementioned paper or computerised register at the farm or other premises for at least two years [2].

LMD environmental impacts are also widely examined by the scientific literature (e.g., [4,5]). The research on fertiliser use, including LMD, highlights its environmental impacts, especially concerning nitrogen and phosphorus runoff contaminating water sources [6,7,8,9,10]. Additionally, the volatilization of excess nitrogen, predominantly in the form of ammonia gas, could significantly compromise air quality.

LMD regulation and handling is often limited to a local scale (e.g., [11,12,13]); differences in LMD production, storage, processing, and spreading can be encountered even within the same region [14,15,16,17]. Surveys conducted in the United States of America, and in particular in the Midwest and California, on a large number of farms [12,14,16] provide more detailed information on LMD management. One example concerns the distribution phase: even when complying with permit requirements, it is possible for operators to over-apply LMD nutrients beyond the needs of the crops [11]. A further complication, highlighted in Shea et al., 2022 [18], and Pedrayes et al., 2023 [19], consists of finding public data on farms and on their spreading plans.

In spite of regulations, spreading that does not comply with the requirements of good distribution practice has been observed. It has been noticed, through satellite images, that some soils show persistent traces of LMD for a continuous period, certainly lasting several days, probably in contravention of the regulations of burying within 24 h of carrying out the practice. Therefore, investigating Earth Observation (EO)-based solutions through the use of satellite data, could significantly improve the identification of amended fields and their monitoring. However, the literature on the use of EO, referring mainly to multispectral satellite data, for LMD detection, is still limited. A relevant study is that of Yang et al., 2002 [20], in which, to analyse multispectral data, decision trees are used to distinguish between organic LMD and chemical fertilisers in two different types of crops. In the study, aerial multispectral images with a spatial resolution of two metres were employed [20]. Usually, aircraft imagery has a greater spatial resolution than satellite imagery but its revisit time is too long for a monitoring system; while the spectral resolution of airborne sensors is often lower compared to satellite data. However, this varies depending on the type of sensor installed on the aircraft. In addition, the high costs for several flights over time and the necessary equipment must be taken into account.

Further studies focused on LMD visualisation use multispectral images acquired by older satellites such as Landsat 5 and 7 [21], which have a smaller number of multispectral bands available with respect to those of more modern satellites such as Landsat 8 and 9 or Sentinel-2, the most widely used [13,22,23,24]. By employing a wider range of wavelengths (bands), modern satellites can distinguish different types of land cover with greater accuracy. Several works [13,20,23,25] agree that bands based on ShortWave Infrared (SWIR) wavelengths are the most suitable for fertiliser/nitrogen detection. This includes vegetation indices that are also composed of reflectance values specific to the SWIR bands. Wavelength analysis in the SWIR range also represents a significant indicator for land cover and land use [26,27,28].

In the recent years Dodin et al., 2021, proposed four spectral indices, named the Exogenous Organic Matter Index (EOMI), (EOMI1, EOMI2, EOMI3, and EOMI4), able to detect green waste compost and livestock manure on sample fields located in the Versailles plain, France [13]. A follow-up study carried out in northern Spain [19] proposed an automated monitoring system for detecting freshly manured fields using machine learning methods also through Sentinel-2 satellite images analysis. More than fifty indicators, including the four EOMI indices and several vegetation indices, have been implemented in order to isolate the best variables for the classification of the manured fields.

Some studies have employed Synthetic Aperture Radar (SAR) for LMD detection by analysing soil moisture levels [18]. Furthermore, machine learning algorithms have been utilised to identify fertilisers with high nitrogen and phosphorus contents [25,29].

Compared with the cases seen in the literature, which are more focused on the detection of nitrates and the state of vegetation, this study aims to detect LMD spreading on bare soil without using specific test areas but by carrying out sample surveys. Detecting and monitoring the LMD spread-affected fields without any previous indications on the fertilisation practices can be challenging, due to the variations in the phenomenon both in the spatial and in the temporal scale. The proposed methodology aims to improve the detection of LMD in large territories, utilising freely available Sentinel-2 satellite data. This aim is achieved through the development of a new spectral index, the Manure Spectral Index (MSI), specifically designed for the study area, focusing on spectral bands where LMD shows more evident variations. As Dodin et al., 2021, and Pedrayes et al., 2023, suggest, the implementation of specifically designed indices could improve the accuracy of such classification processes [13,19]. Ultimately, specifically developed spectral indices could be implemented in the development of LMD monitoring tools. In this regard, Tornato et al., 2023 [1] already applied the results of the spectral index presented in this work to create a Geographic Information System (GIS) tool capable of estimating susceptibility to LMD spreading within the Emilia-Romagna region.

## 2. Materials and Methods

**Figure 1:**
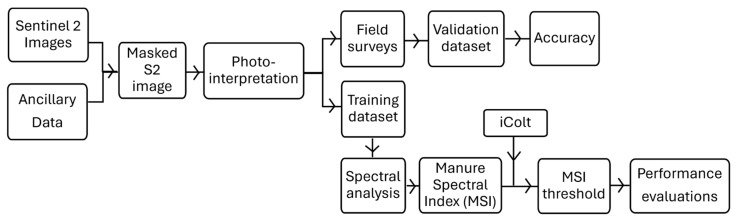
This figure shows the schematic representation of the workflow adopted in this study to create the Manure Spectral Index.

This paper proposes the development phases and application of a new spectral index capable of detecting soils affected by the spreading of LMD. The main steps of the process are illustrated in Figure 1.

### 2.1. Study Area

**Figure 2:**
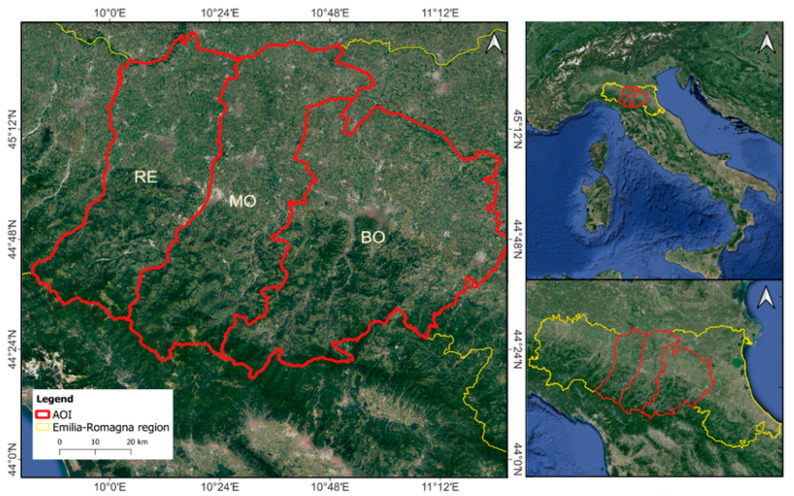
This figure shows the Area of Interest, AOI, in the red lines, corresponding to the provinces of Reggio-Emilia (RE), Modena (MO), and Bologna (BO). The AOI is located within the Emilia-Romagna region, Italy (yellow line). Reference System: WGS84.

The selected study area covers 236,344 hectares of agricultural fields located in the provinces of Reggio Emilia, Modena, and Bologna within the Emilia-Romagna Region (Italy). See Figure 2. Only the lowland area up to a maximum of 150 m above the sea level (a.s.l.) was considered for the research, with an average altitude of 42.6 m a.s.l.

**Figure 3:**
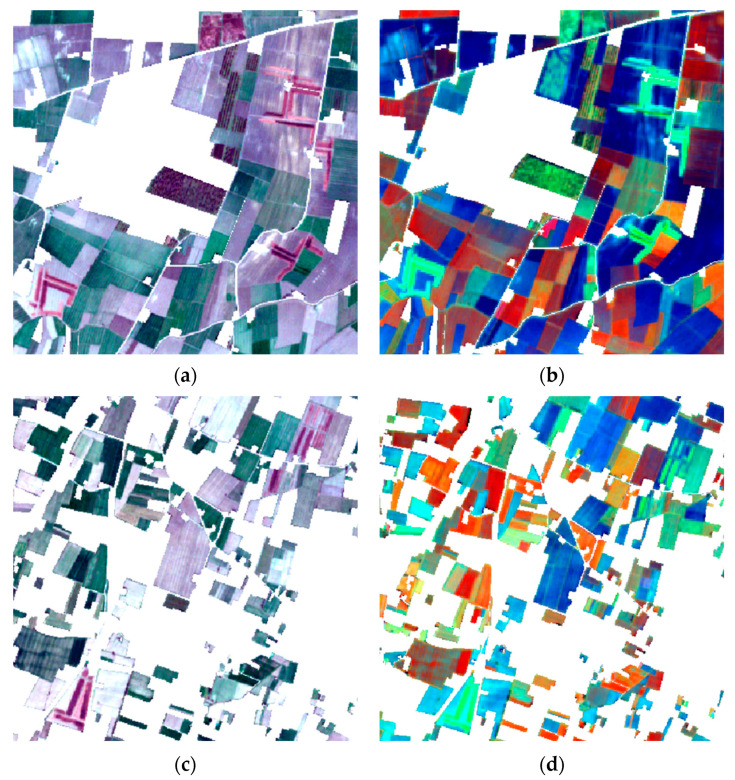
These figures show two details of the 22 February 2022 acquisition, pre-processed with the raster mask, inspected through true-colour and false-colour band combinations: (**a**,**c**) a true-colour combination; (**b**,**d**) a false-colour combination. In the true-colour images (**a**,**c**), manured fields are visible as dark brown stripes, while in the false-colour images (**b**,**d**), the spreading is recognizable by a bright green tint.

The region shows a high density of agricultural activities; in the study area, there are 2036 farms [30] and 102 biodigesters (updated to 2020) [31], whose processing residues have characteristics partly similar to LMD. The substantial number of these agricultural facilities and their subsequent need for LMD spillage make this area suitable for our investigation. The amount of manure and anaerobic digestates available and intended for farming reuse is particularly important during sensitive periods related to environmental constraints: in October, when LMD is spread for winter crops; between November and February, during the LMD ban; and in March, when the soil is prepared for new crops. The study area shows repeated spillages of animal (manure) and vegetable (digestate) effluents over time. These agronomic practices cause peculiar shapes to be created on the land, such as long dark-coloured stripes at the edge of the field, clearly identifiable both from ground surveys and from satellite images acquisitions (Figure 3).

No specific test sites were identified within the examined region, due to the variations of the fertilisation practice both in the spatial and in the temporal scale. In this regard, both LMD spread-affected fields, probably buried within the satellite revisiting time (not found in the subsequent satellite acquisition, referring to Sentinel-2 data with revisit times of 5 days), and LMD spreads persisting for a longer time range have been observed.

The selection of study areas has been executed by identifying fertilised fields through photo-interpretation activity. For this task, four Sentinel-2 images with 10 m of spatial resolution, constituting the training dataset (as shown later), were manually inspected with a false-colour combination obtained by applying an RGB display in which the Near-Infrared (NIR), SWIR1, and RED bands are used in this order. A sample of 9576 pure pixels on the spreading class, widely distributed over the study area, were collected as training dataset.

Five field investigation campaigns have been conducted on the following dates: 22 February 2022, 31 October 2022, 17 February 2023, 1 June 2023, and 10 September 2023. The field surveys were led both to evaluate photo-interpretation performances and to retrieve ground truth fields for an independent validation dataset. This dataset consists of 6125 pixels detected in four acquisition dates only on surveyed fields. Ground truth areas are located in the municipalities of Minerbio (Bologna, Italy), San Giovanni in Persiceto (Bologna, Italy), Mirandola (Modena, Italy), and Rio Saliceto (Reggio Emilia, Italy). See Figure 2.

### 2.2. Satellite Data

Sentinel-2 is an EO satellite of the European Union’s Copernicus Programme, equipped with a multispectral optical sensor called the MultiSpectral Instrument (MSI), and with an average revisit time of five days, due to the presence in orbit of two twin satellites: S2-A, launched in 2015, and S2-B, launched in 2017. The acquisition spectral range is composed of 13 bands characterised by different spatial resolutions: four bands of 10 m, six bands of 20 m, and three bands with spatial resolutions of 60 m [32]. Copernicus satellite acquisitions are freely accessible within specific repositories, characterised by various levels of pre-processing: Level 0, Level 1A, Level 1B, Level 1C, and Level 2A (only the latter two are available for free) [33].

In this study, a pre-existing dataset provided by the regional Arpae (Agency for Environmental Prevention and Energy of Emilia-Romagna) was used, containing pre-processed Level 1C acquisitions from the Sentinel 2 satellite. These images are provided with a radiometric correction to the Top Of Atmosphere (TOA) and a geometric correction, which includes the projection into the UTM/WGS84 cartographic reference system [34,35,36,37]. The use of Sentinel 2 satellite imagery Level 1C ensures continuity and comparability with the monitoring tools used within the regional Arpae. We therefore wanted to specifically develop the MSI index for satellite images without Bottom Of Atmospheric (BOA) correction to optimize the result and evaluate the ability of the index to detect LMD using this type of dataset.

Twelve S2 satellite acquisitions (Table 1), characterised by cloud coverage of less than 6%, have been selected. For each acquisition, the 20 m and 60 m resolution spectral bands have been resampled at 10 m using the nearest neighbour algorithm, and the reflectance has been computed. All the processing operations have been executed using the software ENVI version 5.6.

### 2.3. Ancillary Data

Precipitation, solar radiation, and soil water content data were used during the study of the spectral behaviour of the LMD. These were obtained from the CRITERIA modelling system developed by the Arpae Hydro-Meteo-Climate Service for soil moisture estimation [38].

In addition, regional cartography was used to focus the search only on fields potentially affected by the spreading practice: a Soil Map [39] of the Emilia-Romagna Region for the soil’s characterization; the Land Use Cartography [39] and ISPRA (Italian Institute for Environmental Protection and Research) Report on Land Consumption [40], both at a 1:10,000 scale, to exclude urban and non-agricultural areas; an Early Crop Map (scale 1:50,000) [41] to exclude meadows and alfalfa. These will be used in the pre-processing (see the workflow in Figure 1) of all Sentinel 2 acquisitions used in the project [42].

### 2.4. LMD Spectral Analysis

In order to better evaluate spray field-affected zones, only the agricultural land cover class has been considered. Thus, both the Emilia-Romagna Land Use and Land Cover cartography, and the ISPRA Soil Consumption Report have been employed. A raster mask file was created in order to remove the land cover classes deemed not useful for the purpose of the survey (e.g., build-up, water bodies, infrastructures, etc.).

A preliminary phase of the detection of LMD-affected fields for the entire study area has been carried out through photo-interpretation. In particular, the 22 February 2022 acquisition, pre-processed with the raster mask, was inspected through true-colour and false-colour band combinations. Utilising an RGB visualisation where NIR, SWIR1, and RED bands are used in this order, the manured spreading areas are shown in bright green, as depicted in Figure 3b,d. A field campaign in two of the areas that exhibited more candidate-spilled regions was conducted on 22 February 2022. The operations have been carried out in proximity (max. 1 day of difference) to the satellite acquisition date in order to confirm the photo-interpretation results.

Since the data collected from field inspections, preliminarily compared with the respective satellite data, confirmed the adequacy of the proposed methodology, additional S2 satellite images have been selected. In particular, three acquisitions on different seasons have been chosen to evaluate variations in the spectral response. The investigation dates were 13 May 2022, 6 August 2022, and 4 November 2022—corresponding to the spring, summer, and autumn seasons, respectively. For each of the four multispectral images, a training set including three different classes (LMD, bare soil, and vegetated crops) has been created (Section 3.1).

The Jeffries-Matusita, Transformed Divergence statistical test [43] has been computed on the regions of interest (ROIs) composing the training dataset, to verify the separability of the classes involved, considering the different possible selections of characteristics (in this case, depending on the different multispectral bands available). Values above 1.9 demonstrate high separability while a value below 1 indicates poor separability (Section 3.1).

Finally, the spectral signature has been computed over the training set, analysing the variations in the spectral response, defined and validated, over the above mentioned classes (Section 3.1).

Further investigations on the temporal evolution of LMD spectral signatures have been taken into account. For this purpose, three manured spreading fields within the AOI, located in the Mirandola municipality (Modena, Italy), were selected; those areas appear to be not tilled for an extended time interval after the spreading event (approximately one month). For each field, a time-series of contiguous cloud-free satellite acquisitions (independent from the training set) in which LMD spillage is visible were examined (Table 1). Thus, the time series have been separately analysed in order to test whether changes in the reflectance values have occurred (Section 3.1).

### 2.5. Spectral Comparison between Bare Soil and LMD

**Figure 4:**
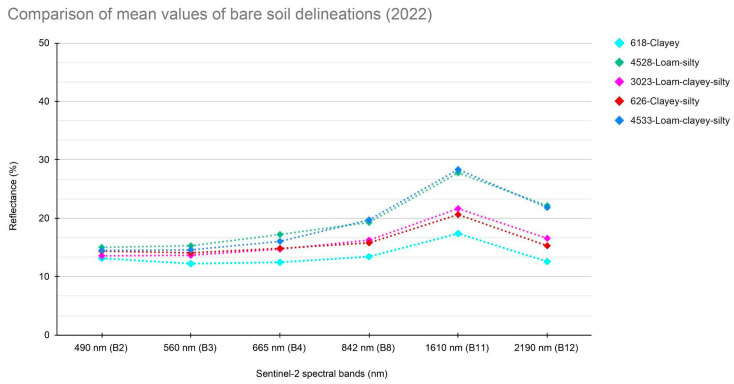
This graph shows the comparison of spectral signatures of the main bare soil delineations of the AOI represented by their respective mean values.

A comparison between the spectral signature of bare soil in the areas affected by spreading and the spectral signature of the LMD was necessary to discriminate among them. First, a time series of six Sentinel-2 acquisitions covering the interval between 6 August 2022 and 5 September 2022 was selected (Table 1). Using the Soil Map of the Emilia-Romagna Region, main soil delineations were identified in which the fields with spreading in the study area are included: clayey soil, silty-clayey soil, loamy-clay soil, loam-silty soil, and loam-clayey-silty soil (Figure 4). For each of the main delineations, sample pixels were extracted and the spectral signature on the reference dates was calculated. The summer period was chosen to acquire reflectance values with little influence on soil water content. In addition, the Criteria model dataset [38] was used to estimate soil moisture, taking into account parameters such as solar radiation, precipitation, and soil water content. This methodology made it possible to extract the average spectral signature of the bare dry soil for each delineation; this data was then compared with the spectral signature of the LMD (Section 3.2).

### 2.6. Spectral Index Development

The development of spectral indices specifically tailored for LMD can still represent an essential issue in the field of spreading monitoring and detection. The above-mentioned spectral characterization of the manured spreading plots has been primarily considered in the development phase of the proposed spectral index. Furthermore, the spectral bands composing the EOMI3 index (Equation (1)) [13] have been particularly taken into account, as the authors suggest a higher correlation between those spectral bands and fertiliser products.

(1) $$\mathrm{EOMI}3=\frac{\left(B11-B8A)+(B12-B4\right)}{B11+B8A+B12+B4}$$

The Manure Spectral Index, MSI, (Equation (1)) is calculated through the ratio between the reflectance values of the two more responsive bands (B11 and B12) and the reflectance value of the visible band that absorbs less radiation from the LMD. The infrared band (B8) has been subtracted in order to mitigate the vegetation contribution.

(2) $$\mathrm{MSI}=\frac{(B11+B12)-B8}{B4}$$

Compared with EOMI3, the MSI maximises the weight of SWIR bands where the spectral signature of LMD peaks.

### 2.7. MSI Threshold Identification

For the detection of LMD, a threshold value has been defined. For this purpose, the mean index values of the non-manured spreading fields and the index rates extracted from the training dataset have been compared. The analysis of the agricultural fields has been performed using the Arpae iColt map, which provides a detailed, accurate, and annually updated crop classification [44] present in the territory of Emilia-Romagna Region. Thus, from the 2022 iColt map, the autumn–winter crops, the summer herbaceous crops, and the multi-annual crops classes (considered as the most representative samples for the study area) have been extracted. For each of the training dates, an NDVI (Normalised Difference Vegetation Index) [45] fixed threshold of 0.30 was defined, thus enabling a further separation of the three above-mentioned classes between the presence or absence of vegetation at the examined time. The resulting dataset was, therefore, composed of six different classes: autumn–winter grasses on bare soil, alfalfa and meadows on bare soil, summer grasses on bare soil, autumn–winter grasses on vegetated soil, alfalfa and meadows on vegetated soil, and summery grasses on vegetated soil. A reduction of the noise component was achieved by removing homogeneous fields with an area lower than five hectares. The proposed analysis aimed at monitoring if the spectral index values presented a similar range independently from the cultural practice, or if differences caused by variations in crop management (i.e., the enhanced frequency of crop residues) have occurred.

A boxplot analysis between the index values recorded on the manured spreading fields and in the six iColt classes enabled us to identify the threshold over the four training satellite acquisitions (Section 3.3). The total examined area is about 130,000 hectares, three orders of magnitude greater than the spreading training dataset. This improvement has allowed us to better understand the index value distribution over the vegetated fields and the bare soil classes, increasing the robustness of threshold selection methodology and the ability of the index to discriminate between LMD and other classes.

### 2.8. Performance Evaluation and Accuracy Methods

#### 2.8.1. Correlation Analysis of the MSI, MNDWI, and NDVI

**Figure 5:**
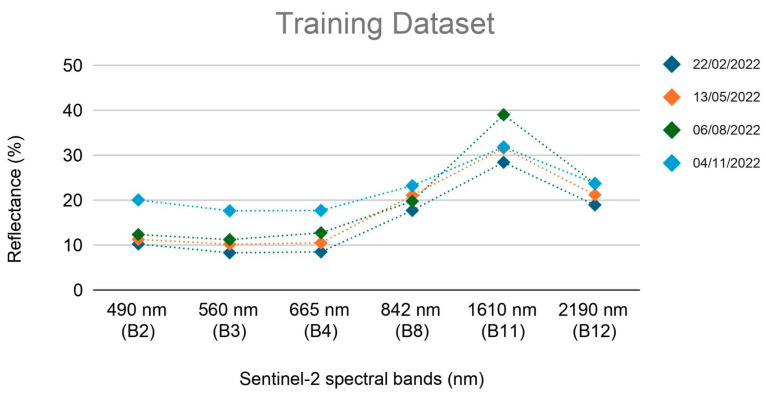
LMD spectral signatures for each acquisition of the training dataset.

Variations in the index values attributable to other processes than LMD have also been considered. The investigation primarily focused on soil moisture changes due to the established inverse relationship between water content and reflectance values in the SWIR bands [46]. Since enhanced SWIR values over the manured fields have been observed (e.g., Figure 5), assessing the influence of water content has been deemed relevant. Thus, the correlation between the MNDWI index [47] and the MSI index has been measured on the training dataset (Section 3.4.1). The soil moisture level has been also retrieved from the Arpae CRITERIA 1D model [38] for comparison. Later, vegetation influence on index values has been investigated; scatter plots correlating the NDVI with the spectral index values have been computed (Section 3.4.1).

An additional investigation on the validation dataset was carried out to understand whether some variations in false-colour visualisation could be ascribed to an influence of soil moisture or vegetation. Therefore, the validation dataset has been subdivided into two sub-classes considering both false-colour photo-interpretation and variations in the spectral signature and the observations from the field campaigns. The first sub-class (SC-h) includes the fields characterised by a high spectral response in the SWIR region and an optimal detection on photo-interpretation, while the second one (SC-l) comprises the regions with the opposite attributes (Section 3.4.1). The index rates of the sub-classes were then compared with the values derived from the MNDWI and NDVI, concerning humidity and vegetation, respectively.

#### 2.8.2. The OLR Tests

The overlapping rate test (OLR) has been computed over the validation dataset. The proposed indicator has been successfully employed by Nguyen et al., 2021 [43], in the assessment of the capability of spectral indices to effectively separate between different classes (Section 3.4.2). The OLR for the LMD class has been computed using Equation (3):

(3) $$OLR=\left(\frac{\sum_{i=1}^{N_{tot}}k_{i}}{N_{tot}}\right)$$

where *i* = 1, 2, … *N_tot_* varies over the measured values, and *N_tot_* corresponds to the total number. The value of the coefficient k depends on the index rates within the LMD class. The coefficient equals 0 when the index rates in the LMD class are greater than the maximum value among both the vegetation and soil classes. Conversely, when the LMD rates fall below this threshold, *k* equals 1. Thus, the OLR expresses the fraction or percentage of LMD values overlapping with the bare soil and vegetation classes.

The overlapping rates have been analysed both between the three above-mentioned classes and in comparison with the EOMI3 index [13].

#### 2.8.3. Accuracy

The accuracy assessment of the index performances using a simple threshold value have been carried out by computing confusion matrices and the kappa coefficient [48] (Section 3.4.3). Such operations have been executed on the validation dataset only, in order to ensure statistical independence. In addition to the LMD class, a set of pixels of the vegetated fields and of the bare soil classes have been added for the evaluation of false positives.

## 3. Results

### 3.1. LMD Spectral Analysis

The Jeffries-Matusita, Transformed Divergence statistical test [43] outcomes are reported in Table 2 and suggest a good separability rate for the whole training dataset, therefore supporting the suitability of the dataset for the proposed analysis. Values above 1.9 demonstrate high separability while a value below 1 indicates poor separability (Section 2.4).

Over the manured spreading regions, an increase of the reflectance values in the SWIR region, corresponding to the bands 11 and 12 of S2 have been observed (Figure 5). In particular, B11 presents the highest increase with respect to the bare soil class. Furthermore, a quite consistent decrease is also noticed across the visible region, coinciding with the bands 2, 3, and 4.

A comparison between the spectral signatures (Figure 5) of the satellite acquisition composing the training dataset suggests a coherent spectral response throughout the seasonal cycle.

**Figure 6:**
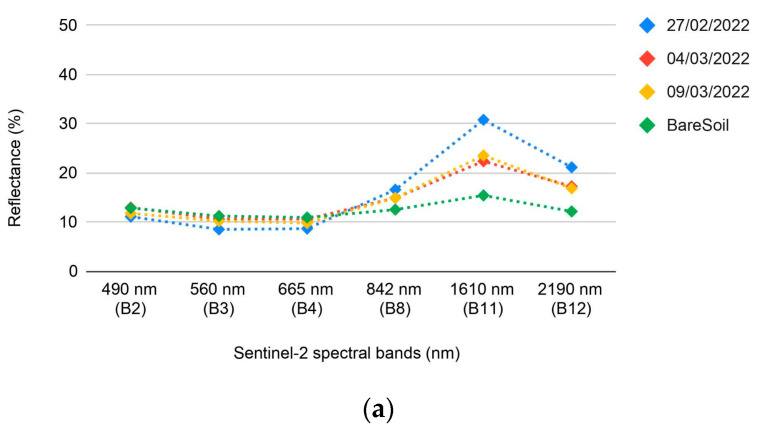

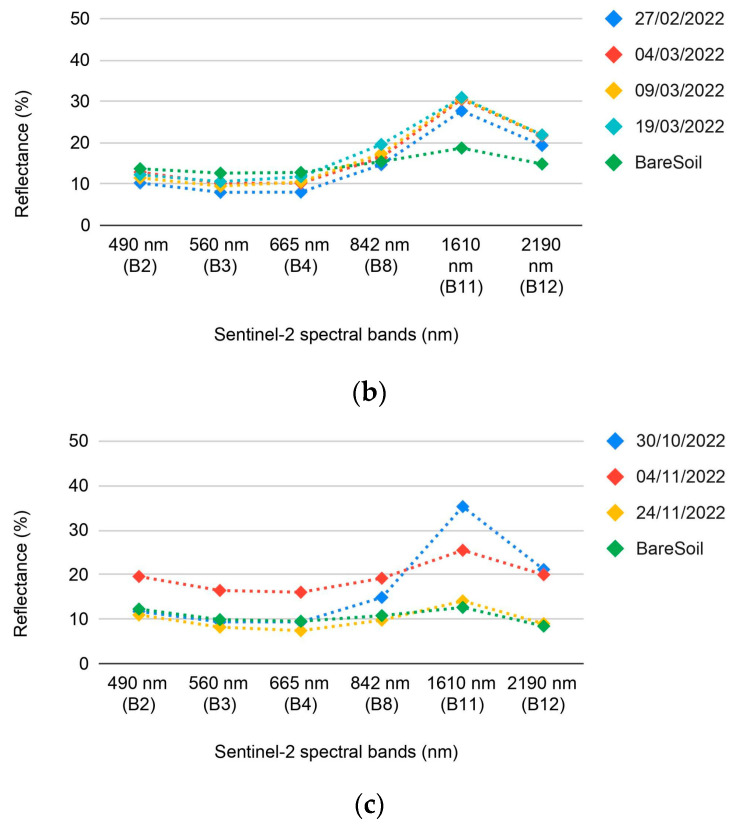
Graphs (**a**–**c**) show three examples of the evolution of the LMD spectral signatures. Each time series is referring to a single field located in the Mirandola (MO) municipality.

Analysis of spectral signatures from the three presumed non-tilled fields reveals a variable decrease in reflectance in the SWIR bands, particularly within B11, starting from the hypothesised spreading time (Figure 6). Furthermore, a slight increase in the visible bands has been noticed. It could be suggested that, further from the spillage time, reflectance values of the LMD tend to be similar to the bare soil ones.

### 3.2. Spectral Comparison between Bare Soil and LMD

**Figure 7:**
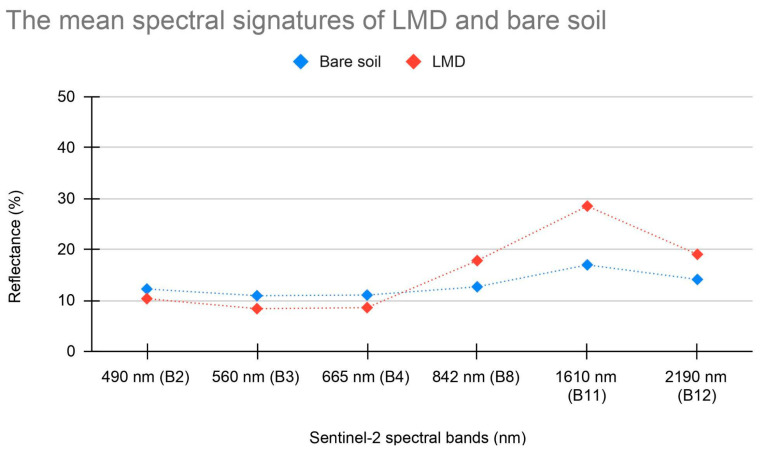
Comparison between the mean spectral signatures of LMD and bare soil.

A comparison of the spectral signatures of bare and spreading soils (Figure 7) showed that the reflectance values in band 11 of the LMD are typically much higher than those of the bare soil detected in the summer period in the same soil delineation. This aligns with the results of the LMD spectral analysis described above. Additionally, the reflectance values in band 11 in the presence of dry soil are, on average, higher than in the case of wet soil conditions.

### 3.3. Manure Spectral Index and Threshold Identification

From the computation of the proposed spectral index over the training dataset, higher index rates have been detected over the LMD fields. Therefore, a good agreement between the photo-interpretation outcomes and the index rates over the manured spreading areas can be supported.

**Figure 8:**
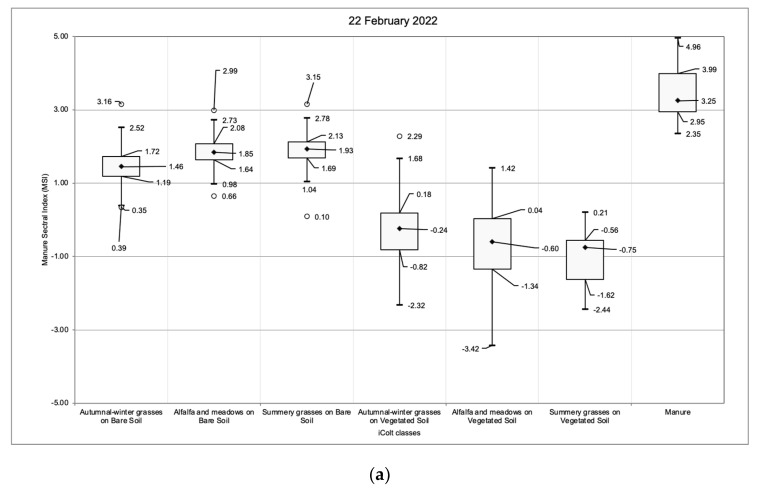

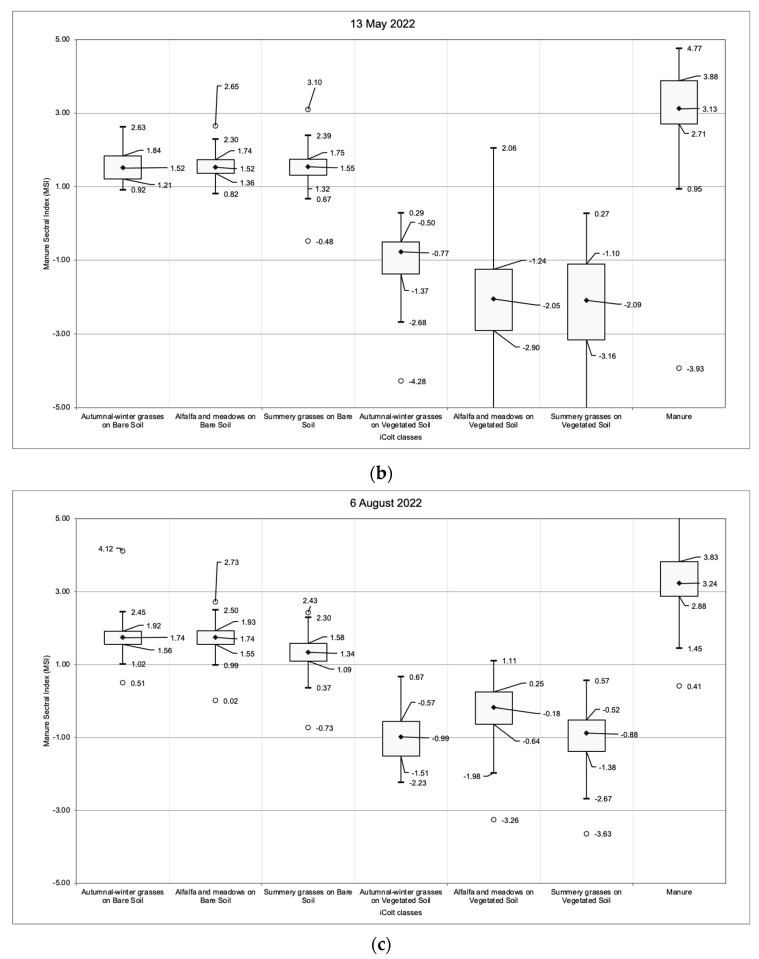

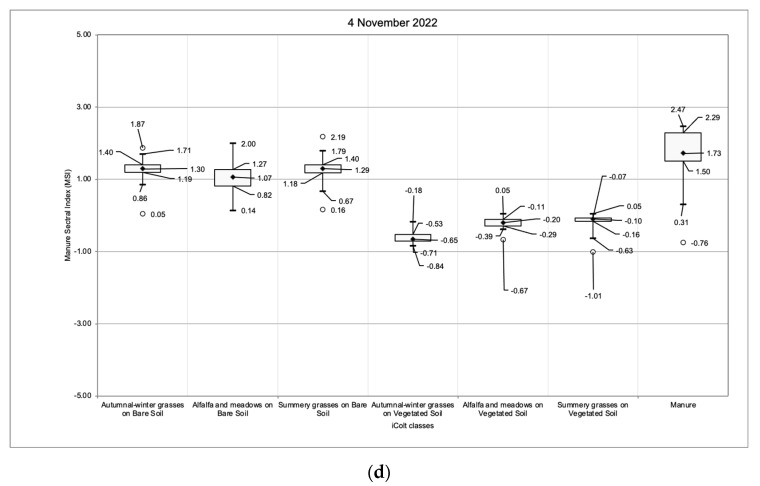
These graphs show the results of boxplot analysis with respect to the sub-classes derived from the iColt classification. Training datasets: 22 February 2022 (**a**), 12 May 2022 (**b**), 6 August 2022 (**c**), and 4 November 2022 (**d**). On the *x*-axis are shown the LMD class and the iColt subclasses: autumn–winter grasses, alfalfa and meadows, and summery grasses; each one on bare soil and vegetated soil conditions. In the plots, squares represent the interquartile range (IQR), while the error bars were computed in accordance with the Tukey fences rule. The black diamonds correspond to the median rate, the circles correspond to the outliers.

The results of the boxplot analysis (Figure 8) performed on the entire study area provided not only a better estimation of the best threshold for the spectral index, but also a deeper understanding of the distribution of index rates on the bare soil and vegetated crop classes. The former class presents values slightly lower than those of the LMD area, while the latter exhibits a substantial decrease in the index rates.

With respect to the sub-classes derived from the iColt classification, no substantial trends in the index values have been observed between the crop types (Figure 8). However, the variations depending on the vegetation proportion seem to confirm the tendency recorded on the training set.

The first quartile of the LMD class, excluding outliers, exceeds the upper whisker of both the bare soil and vegetated field classes, potentially indicating a suitable threshold for classification. Values exceeding the error bars calculated using the Tukey fences rule [49] have been excluded. The outliers thus defined correspond to 1% of the distribution. Only the acquisition of 4 November 2022 (Figure 8d) shows a wide reduction in the index rates in all of the classes probably due to the high soil moisture rate; nevertheless, LMD plots exhibit higher index values with respect to the other classes (even if this is with a greater error rate).

### 3.4. Performance and Evaluation Accuracy Results

#### 3.4.1. Correlation Analysis of the MSI, MNDWI, and NDVI

**Figure 9:**
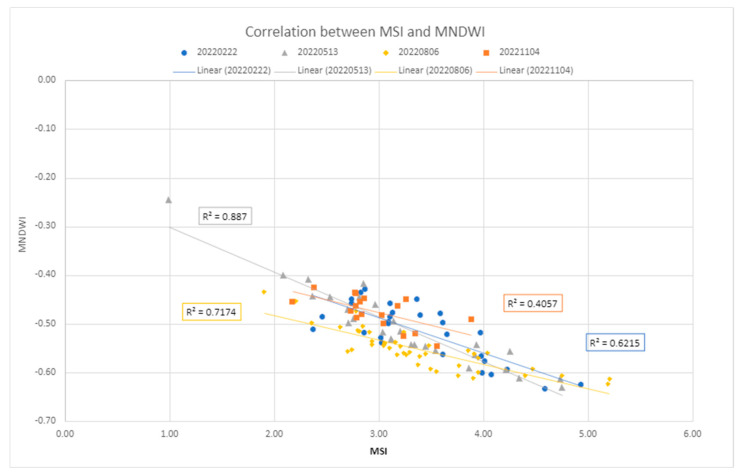
This graph shows the correlation between the moisture index, MNDWI, and the Manure Spectral Index (MSI). The MNDWI values range from −1.0 to +1.0. The value of −1.0 corresponds to the dry surface, while the value of +1 corresponds to the water surface.

A comparison of MSI values with MNDWI values shows a good linear correlation of an inversely proportional nature (Figure 9): as moisture values increase, MSI index values decrease. This is evident from the November 4 image, whose soil, according to CRITERIA data [38], is classified as water-saturated: low MSI index values correspond to high MNDWI index moisture values.

**Figure 10:**
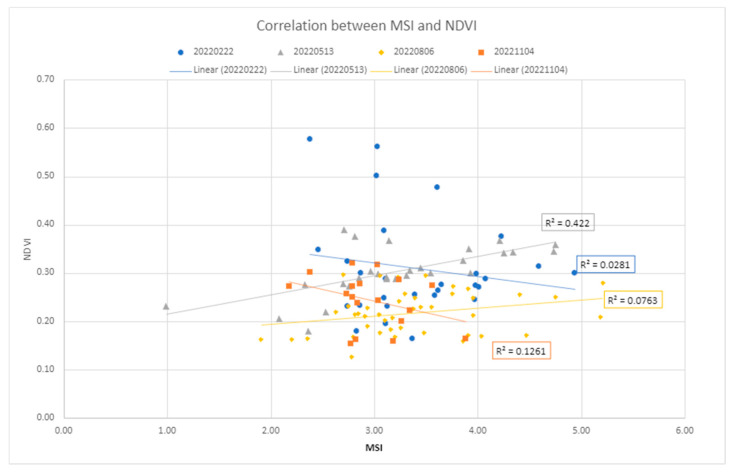
Correlation between the vegetation index, NDVI, and the Manure Spectral Index (MSI). The NDVI values range from +1.0 to −1.0. Generally, below 0.2, there is no vegetation and above 0.5, there is dense vegetation; between 0.2 and 0.5, sparse vegetation is present.

The computation of the MSI over the training dataset showed that the vegetation class exhibits low MSI values. This information prompted us to verify the correlation. However, the comparison of MSI values with NDVI values did not produce any particular results (Figure 10), as there is no visible correlation between the two variables.

**Figure 11:**
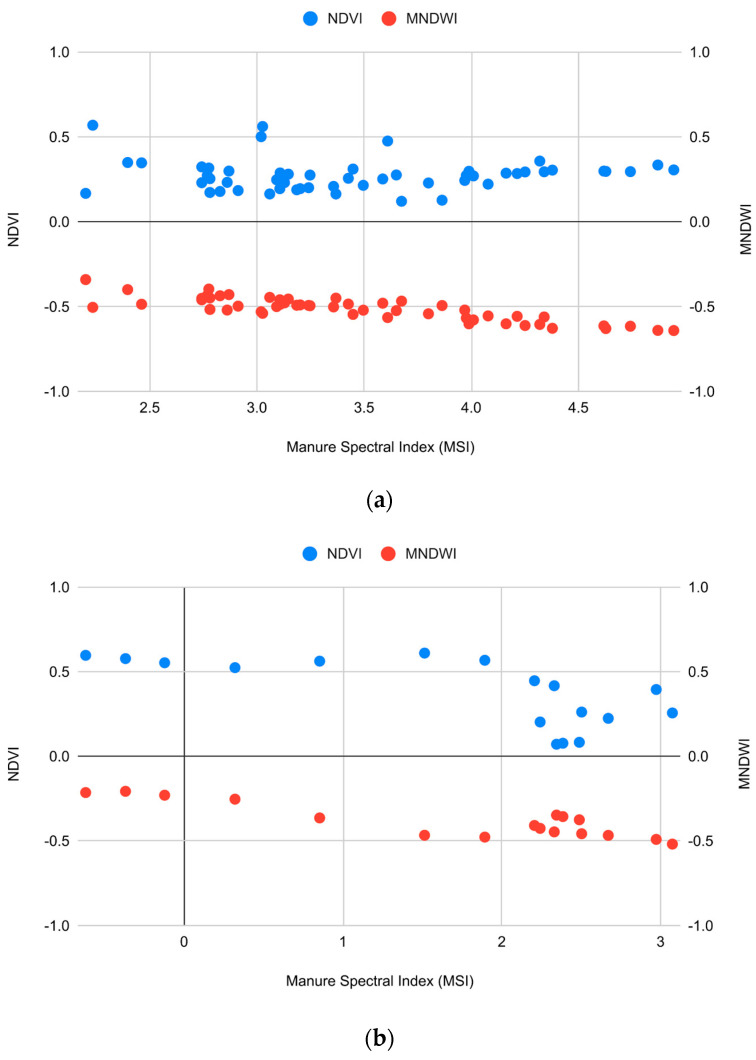
Distribution of the MSI index rates in the two sub-classes, the SC h class (**a**) and the SC-l class (**b**), in relation to the MDWI and NDVI indices, the right and left axis, respectively.

Ultimately, the distribution of MSI values depending on the MNDWI and NDVI variables in the SC h and SC-l classes have been investigated. For the first class, an almost constant linear trend between the variables has been observed; however, few sparsely distributed points can be distinguished (Figure 11a). In the second graph (Figure 11b), relative to the SC-l class, a correspondence between high values of the MNDWI and low values of the MSI index could be hypothesised. It can also be suggested that the presence of vegetation (NDVI > 0.50) could reduce MSI values. The presented distributions, in relation to the MNDWI variable, are consistent with the results obtained for the training sets (Figure 9).

#### 3.4.2. The OLR Tests

**Figure 12:**
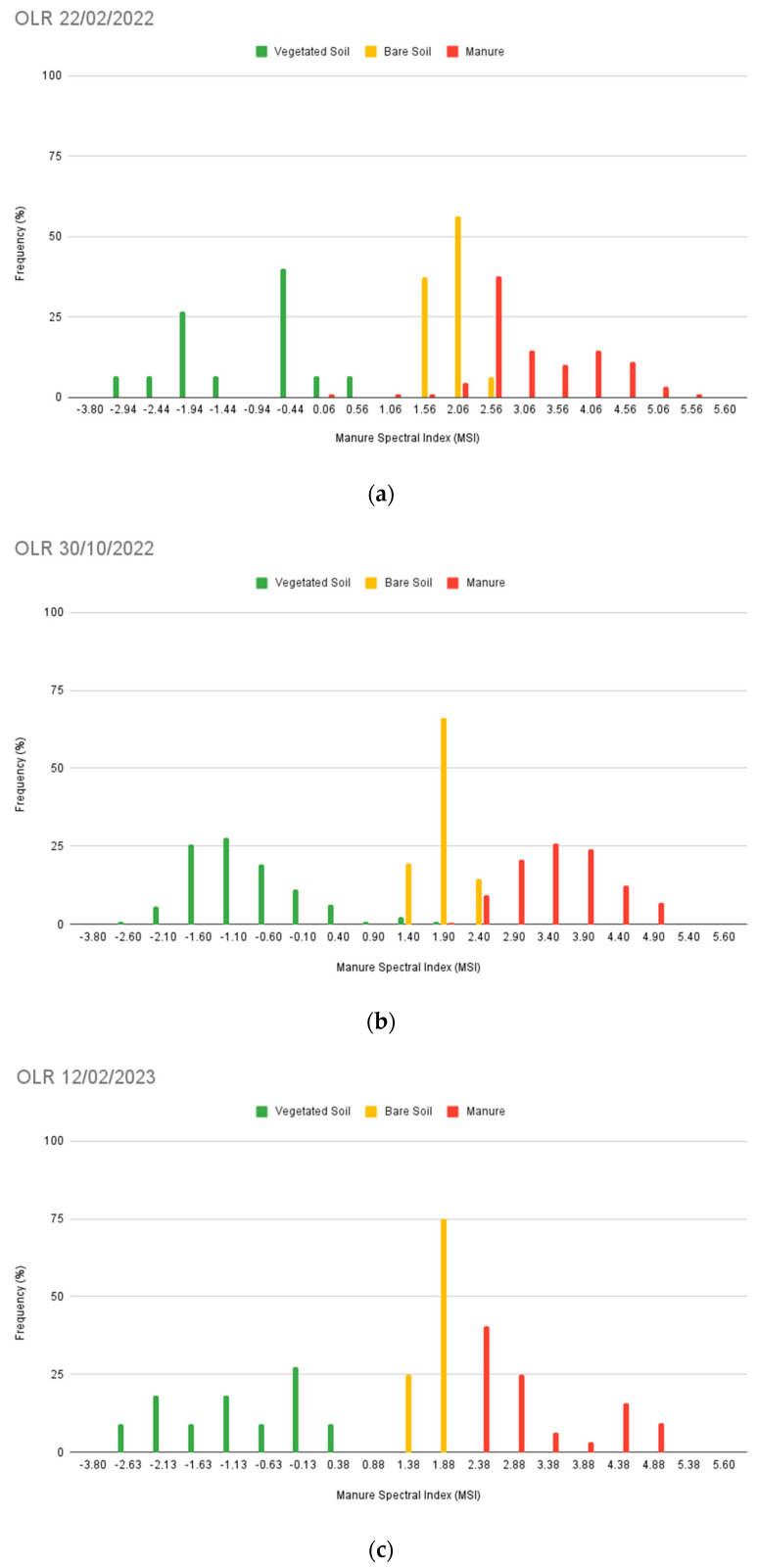

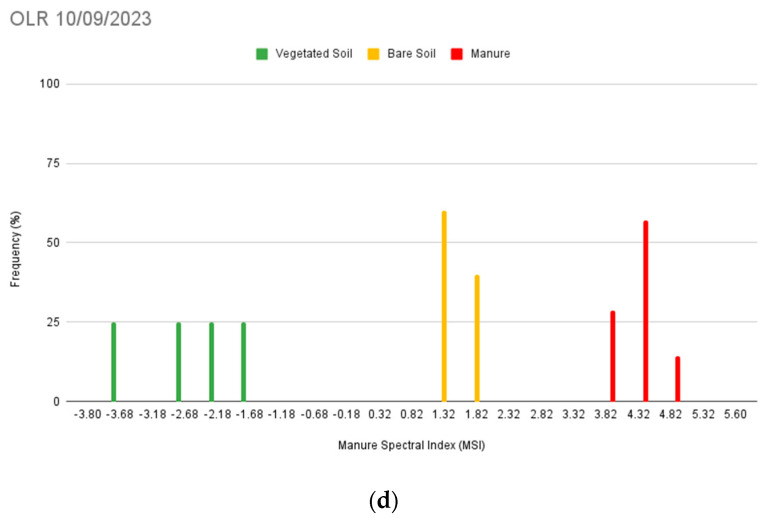
OLR results relative to vegetated soil, bare soil, and manure (LMD) classes for the dates of 22 February 2022 (**a**), 30 October 2022 (**b**), 17 February 2023 (**c**), and 10 September 2023 (**d**).

In addition to the confusion matrix, the OLR test has been measured, as it could strengthen the accuracy assessment procedure. Variable rates, likely affected by the ROI number, have been observed; the higher recorded value is equal to 10.0%, while the lower ones range from 1.3% to 0% (Figure 12). Such outcomes could support the capability of the spectral index to detect LMD.

**Figure 13:**
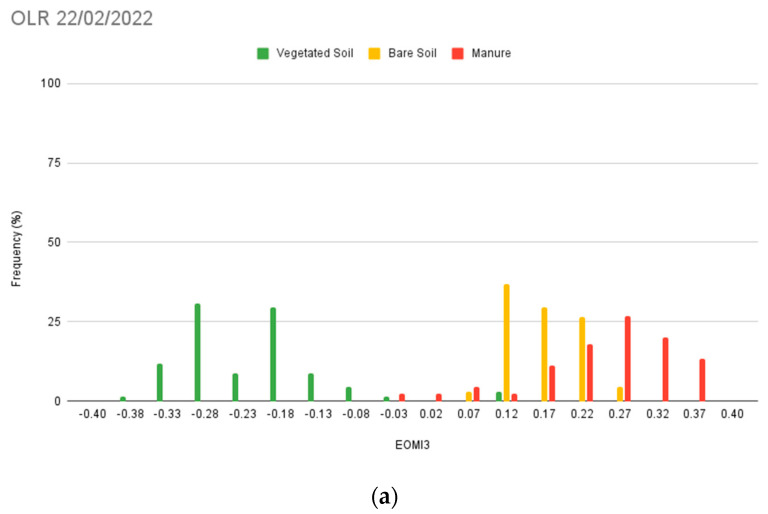

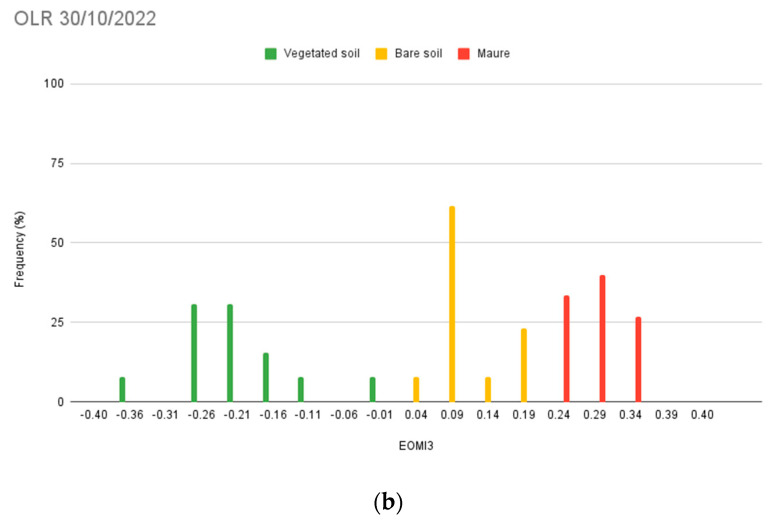
OLR results in two of the four acquisitions of the training dataset: 22 February 2022 (**a**) and 30 October 2022 (**b**). The manure class refers to LMD.

**Figure 14:**
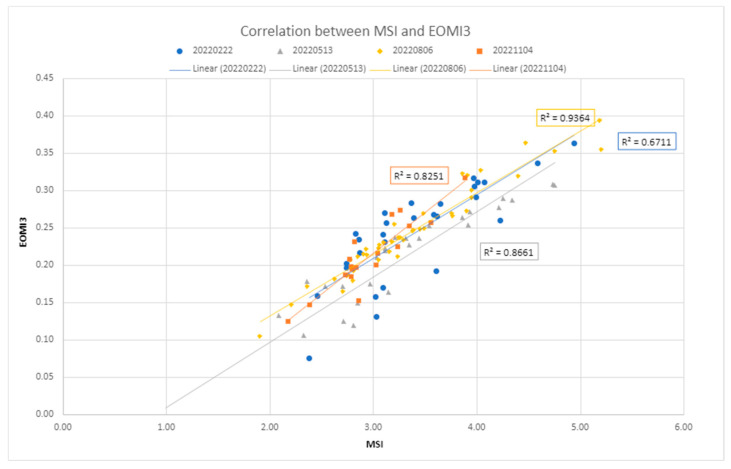
Correlation between the MSI index and the EOMI3 index [12] related to the training dataset.

For comparison, the OLR test has also been executed over the EOMI3 values (Figure 13). Substantial differences have been registered only for the 22 February 2022 acquisition, while on the other elaborations the two rates appear to be coherent. The overlapping rate ranges from 0% to 46.66%, with the lowest value observed in the 30 October 2022 image (Figure 13b). From the correlation between EOMI3 and the MSI, there is a visible consistency between the two indices (Figure 14).

#### 3.4.3. Confusion Matrix

The confusion matrix has been calculated to estimate the accuracy rate of the spectral index in identifying fertilised fields (Table 3). The product accuracy is 62.53% in the LMD class, while the value of the kappa coefficient [50,51] is 0.71; the present rate suggests an agreement between the classification and the groundtruth data according to the interpretation scale proposed by Landis and Koch, 1977 [52]. Furthermore, as can be seen in Table 4, the overall accuracy rate, that also includes the control classes, amounts to 87.93%. The same table shows the details of the statistics referring to the confusion matrix (GroundTruth/MSIindex). In particular, the overall errors, per product and per user, as well as the errors of commission and omission are explained. The values are intended to be percentages and the K coefficient was also reported.

## 4. Discussion

The results of the spectral analysis of LMD are very much in agreement with the scenario presented by Dodin et al., 2021 [13] for the second day after the spreading. The authors report a variable increase in reflectance values in the SWIR band B11 and a decrease in the visible bands (B2, B3, and B4) for both green waste compost and livestock effluent with respect to bare soil. The observations of the spectral signature carried out in the present work agree with Dodin et al., 2021 [13] for the visible bands and B11. Additionally, a slight increase in reflectance in B12 has been registered (Figure 5). As pointed out by Dodin et al., 2021 [13], the spectral response to spreading varies due to both the physical composition of the fertiliser and the temporal distance from the time of spreading. However, such additional information relative to the type and amount of effluent, the precise date of spillage, and the presence of dry vegetation are not easily accessible for the proposed study area, which is a limitation of this work. Despite the scarcity of data, the spectral signature of the LMD class exhibits a consistent decreasing trend over time (Section 3.1). The signature in the SWIR bands maintains a higher reflectance in the LMD spectral region compared to the bare soil signature. Furthermore, manured spreading areas can still be detected several days after the event (approx. 15–25), in the absence of tillage practice. The signature in the SWIR bands maintains a higher reflectance in the LMD spectral region compared to the bare soil signature, although B11 reflectance rates tend to progressively decrease from the hypothesised spreading date onwards (Figure 6a,c). A slight increment of the reflectance values could also be observed in the visible bands (in two of the examined fields) (Figure 6a,b). Although a tentative estimation of the spreading time may be carried out through photo-interpretation, a precise identification of the spreading time with the available data remains challenging. In any case, within the five-day revisit time of Sentinel-2, there may be additional and undetectable spreading events, which limits the reliability of any temporal analysis. Despite the uncertainties affecting the spectral signatures, the relevance of bands 11 and 12 of the SWIR for the development of the MSI index has been demonstrated by the present work (Section 3.1).

The performance of the MSI index on vegetated soils has not been investigated; however, in those areas, some fertilised fields have been detected. By comparing the NDVI and the MSI index, no strong correlation emerges, which may point to the index being applicable to vegetated fields (Section 3.4.1). Future research on the index application in such areas should be kept open.

The accuracy rate resulting from the confusion matrix has been obtained considering a fixed threshold (index rate > 3). While the outcome may vary with the chosen threshold, the OLR test ensured statistical independence by analysing all categorised index values (Section 3.4.2). An improved accuracy rate could be achieved including the MSI within specific classification routines; similar methodologies have already been employed in Pedrayes et al., 2023 [19]. It is also worth mentioning that the MSI index has been designed for the presented study area; different morphologies, soils, and LMD compositions, along with other elements, could potentially affect the index response and its accuracy rate.

In conclusion, a comparison of the performance of the MSI and EOMI3 indices was undertaken to determine the extent of their agreement. Dodin et al., 2021, 2023 [5,13] demonstrated the capability of the EOMI indices for LMD detection. However, the lack of accuracy rates has limited them from performing more detailed comparisons. In the present work, the accuracy of the four EOMI indices [13] relative to the current study area was evaluated; it was determined that EOMI3 performs better and emerged as the most appropriate index for comparative analysis in the context of the current AOI, with an overall accuracy of 48.99% and a kappa coefficient of 0.13.

## 5. Conclusions

In the present study, the development of a spectral index capable of detecting LMD spreading has been presented. The accuracy rate of the MSI index was derived, as well as additional tests on performance assessment (e.g., OLR, vegetation and soil moisture correlation). The resulting accuracy of 62.5% has been considered acceptable for the spectral index. Being aware that the accuracy and the K coefficient do not show very high values in their results, these were still considered sufficient and functional for the method that was implemented. This procedure is designed and will be used as an operational tool for the Arpae, as it proves effective for a rapid preliminary assessment of the territory, subsequently allowing more in-depth and targeted investigations in the critical areas identified by the spectral index. Furthermore, it has been suggested to integrate the MSI within classification models to improve LMD detection. Although we are working to implement this aspect, especially to be able to extend the study area, the primary focus of this paper is to present the index, evaluate its accuracy, and discuss the limitations of the method.

The applied methodology confirmed the suitability of Sentinel-2 acquisitions for the detection of fertilisation practices, in agreement with the scientific literature. By analysing a large-scale area without using specific fields as case studies, this work represents a step forward in the field. The applicability has been tested with sample field surveys. Despite the lack of knowledge on both the specific spreading date and the composition of the fertiliser, the MSI identifies LMD spreading. The use of more extensive time series and access to information on the types of LMD used can certainly lead to the refinement of the method, both in studying the spectral response of LMD and in extending the study area to zones with different characteristics. The study focuses on a specific area; however, as previously mentioned, the MSI application might vary depending on the context.

Although the applicability to vegetated soils remains unexplored, the promising results of this study offer potential for further research. Both this study and previous research highlight the capability of remote sensing in enhancing monitoring techniques for fertilisation practices, emphasising the continued importance of this research area.

## Figures and Tables

**Table 1:** The summary table shows the main characteristics of the Sentinel-2 L1C acquisitions and indicates the role of each one within the process.

Acquisition Date	Application	Soil Condition ^1^	Satellite	Resolution	Cloud Cover%
22 February 2022	Training Dataset	Dry	Sentinel-2 L1C	10 m	≤6
13 May 2022		Dry			
6 August 2022		Dry			
4 November 2022		Moist			
27 February 2022	Manure Spectral Signature Evolution	Dry			
4 March 2022		Dry			
9 March 2022		Dry			
19 March 2022		Dry			
30 October 2022		Dry			
4 November 2022		Moist			
24 November 2022		Wet			
30 October 2022	Validation Dataset and Accuracy	Dry			
22 February 2022		Dry			
12 February 2023		Dry			
10 September 2023		Dry			
6 June 2022	Bare Soil Analysis	Dry			
11 August 2022		Dry			
16 August 2022		Dry			
21 August 2022		Dry			
26 August 2022		Dry			
5 September 2022		Dry			

^1^ Soil condition indications result from the interpretation of precipitation and soil water content data from the CRITERIA modelling system [38].

**Table 2:** Separability test results from the training dataset.

Acquisition Dates	Bare Soil LMD	Vegetated Fields LMD	Bare Soil Vegetated Fields
22 February 2022	1.982	1.997	1.999
13 May 2022	1.987	1.987	1.999
6 August 2022	1.902	1.999	1.999
4 November 2022	1.958	1.999	2.000

**Table 3:** Calculated confusion matrix (GroundTruth/MSIindex). The reported values are intended to be the number of pixels.

		Ground Truth (Pixel)		
		Other Classes	LMD	Total
**MSI Index (Pixels)**	**Other Classes**	10,957	1951	12,908
	**LMD**	0	3256	3256
	**Total**	10,957	5207	14,213

**Table 4:** Details of the statistics referred to in the confusion matrix (GroundTruth/MSIindex).

**Overall Accuracy**	**87.93%**	**K Coefficient**	**0.71**	
	**Product Accuracy**	**User Accuracy**	**Commission**	**Omission**
**LMD**	62.53%	100.00%	0.00%	0.37%

## Data Availability

The data can be requested from the corresponding authors.
